# Molecular detection and identification of hemotropic *Mycoplasma* species in dogs and their ectoparasites in Iran

**DOI:** 10.1038/s41598-024-51173-w

**Published:** 2024-01-05

**Authors:** Kimia Beus, Ali Goudarztalejerdi, Alireza Sazmand

**Affiliations:** Department of Pathobiology, Faculty of Veterinary Medicine, Bu-Ali Sina University, Hamedan, 6517658978 Iran

**Keywords:** Bacteriology, Infectious-disease diagnostics

## Abstract

Hemotropic *Mycoplasma* species are vector-borne bacteria that attach and grow on the surface of erythrocytes in various mammals, yet reports of canine hemoplasmosis in Iran are scarce. The aim of this study was molecular detection and identification of hemoplasmas in the blood of dogs (*n* = 370) from five provinces of Iran and ectoparasites infesting them including *Ctenocephalides canis* and *Pulex irritans* fleas, *Rhipicephalus sanguineus* sensu lato ticks, *Heterodoxus spiniger* lice and *Hippobosca longipennis* keds. Hemotropic *Mycoplasma* spp. pathogens were detected using genus-specific conventional PCRs, and subsequently identified using species-specific PCRs for *Mycoplasma haemocanis* (Mhc), and *Candidatus *Mycoplasma haematoparvum (CMhp). Sanger sequencing was then performed to confirm the species. Correlation of infection and risk factors (geographical area, keeping condition, body condition, sex, age, ectoparasite infestation) were analyzed. In total, 210 dogs (56.7%) were tested PCR-positive for hemotropic *Mycoplasma* spp. Species-specific PCR and sequencing revealed infection with Mhc in 17.8%, with CMhp in 7.02% and co-infection in 31.9% of dogs. Flea infestation, poor body condition, and being older than 3-years-old correlated with hemoplasmosis. In ectoparasites, DNA of hemoplasmas were detected only in fleas i.e. Mhc in *P. irritans*, CMhp in *P. irritans* and *C. canis*, and co-infection in *C. canis*. To our knowledge, this is the first large-scale molecular epidemiology study of canine hemoplasmosis in Iran. Considering the high prevalence of canine hemoplasmosis all over the country including potentially zoonotic CMhp, effective ectoparasite control strategies, regular examination of dogs, successful chemoprophylaxis and public awareness strategies are advocated.

## Introduction

Hemotropic mycoplasmas (syn. hemoplasmas) are Gram-negative, small, pleomorphic and uncultivable bacteria infecting a wide range of domestic and wild mammals including dogs, cats, livestock and rarely humans^1–6^. These obligate epierythrocytic parasites induce persistent asymptomatic intravascular infections in animals; however, acute and chronic hemolytic anemia, depending on host susceptibility and coinfection with other pathogens, lead to lethargy to oncogenesis or death^7^.

In dogs, the most common hemoplasma species are *Mycoplasma haemocanis* (Mhc), and *Candidatus* M. haematoparvum (CMhp) which are present worldwide^1^. These bacteria are mostly known to cause varying degrees of hemolytic anemia, but can also induce fever, apathy, adenopathy, motor incoordination, splenomegaly, anorexia, lethargy, jaundice, dehydration, weight loss, and sudden death^7,8^. Although hemoplasma infections are typically species-specific, in addition to Mhc and CMhp, *M. ovis*^9^, *M. suis*^10^, *Candidatus Mycoplasma haemobos*^11^, *Candidatus* Mycoplasma haemominutum^12^ and *Candidatus* Mycoplasma turicensis^13^ have been detected in dogs. Importantly, infections with CMhp, *M. ovis* and *M. suis* which have been detected in dogs’ blood have been described in human patients^3,14–16^. Transmission routes of hemoplasmas in dogs are matter of debates, but bloodsucking arthropods e.g. fleas and ticks have been suggested to act as vectors^17,18^. Moreover, fighting, intrauterine transfer, transmission through lactation and blood transfusion from apparently healthy carrier dogs have been suggested^19–22^.

The prevalence of hemotropic *Mycoplasma* species in dogs has been reported to be between 1.2^23^ and 54%^24^ in different regions of the world. In particular, previous studies performed in the Middle Eastern countries e.g. Türkiye^22^, Qatar^25^, Saudi Arabia^26^ and Egypt^27^ reported prevalence of canine hemoplasmosis to be 38%, 7.8%, 5.7%, and 17% however, there is shortage of information about the occurrence and species composition of hemoplasmas in dogs of Iran since in the only two previous studies, limited number of animals from two cities (Shiraz and Esfahan) were examined^28,29^. Hence, this study aimed to assess the prevalence and molecular characterization of hemotropic *Mycoplasma* species in apparently healthy dogs and their ectoparasites from five Iranian provinces with different climates.

## Results

### Prevalence of hemoplasmas in dogs

Out of 370 dogs tested, 210 (56.8%, 95% CI 51.9–62.2) scored positive to *Mycoplasma* spp. Species-specific PCR assays revealed infection of 66 dogs with Mhc (17.8%, 95% CI 14.3–21.9), 26 dogs with CMhp (7%, 95% CI 4.6–10). Co-infection with both parasites was observed in 118 dogs (31.9%, 95% CI 27.3–36.5). Infection rate was highest in dogs of Khuzestan (Table 1).

**Table 1:** Risk factors associated with prevalence of *Mycoplasma haemocanis* (Mhc) and *Candidatus M. haematoparvum* (CMhp) in dogs of Iran (*n* = 370) according to different variables.

Variable	No. of PCR positive dog			
	Mhc	CMhp	Mhc and CMhp	Total
Gender				
Male (*n* = 136)	21 (15.44%)	8 (6.0%)	53 (38.97%)	82 (60.29%)
Female (*n* = 234)	45 (19.2%)	18 (7.7%)	65 (27.8%)	128 (54.7%)
Age				
≤ 1 year (*n* = 81)	6 (7.4%)	2 (2.5%)	25 (30.9%)	33 (40.7%)
1–3 year (*n* = 129)	28 (21.7%)	10 (7.8%)	34 (26.4%)	72 (55.8%)
≥ 3 years (*n* = 160)	32 (20%)	14 (8.75%)	59 (36.87%)	105 (65.62%)*
Breed				
Mix (*n* = 263)	53 (20.2%)	18 (6.8%)	92 (35%)	163 (62%)
Great dane (*n* = 28)	5 (17.9%)	3 (10.7%)	9 (32%)	17 (60.6%)
German shepherd (*n* = 19)	1 (5.3%)	2 (10.5%)	3 (15.8%)	6 (31.6%)
Husky (*n* = 15)	2 (13.3%)	0	5 (33.33%)	7 (45.63%)
Other (*n* = 45)	5 (11.11%)	3 (6.66%)	9 (20%)	17 (37.77%)
Body condition score				
Excellent (*n* = 22)	2 (9.1%)	3 (13.6%)	8 (36.4%)	13 (59.1%)
Good (*n* = 185)	33 (17.8%)	15 (8.1%)	61 (33.0%)	109 (58.9%)
Fair (*n* = 66)	17 (25.8%)	1 (1.5%)	21 (31.8%)	39 (59.1%)
Poor (*n* = 46)	11 (23.9%)	4 (8.7%)	17 (37.0%)	32 (69.6%)*
No data (*n* = 51)	3 (5.9%)	3 (5.9%)	11 (21.6%)	17 (33.3%)
Infested with ectoparasites				
Ticks (*n* = 44)	3 (6.8%)	0 (0.0%)	20 (45.5%)	23 (52.3%)
Fleas (*n* = 56)	10 (17.9%)	4 (7.1%)	21 (37.5%)	35 (62.5%)*
Lice (*n* = 10)	1 (10%)	0 (0.0%)	2 (20.0%)	3 (30.0%)
Living condition				
Shelter (*n* = 363)	65 (17.9%)	25 (6.8%)	118 (32.5%)	208 (57.3%)
Pet (*n* = 5)	1 (20%)	0	0	1 (20%)
Stray (*n* = 2)	0	1 (50%)	0	1 (50%)
City				
Hamedan (*n* = 135)	15 (11.1%)	8 (5.9%)	39 (28.9%)	62 (45.9%)
Kermanshah (*n* = 50)	10 (20.0%)	3 (6.0%)	15 (30.0%)	28 (56.0%)
Yazd (*n* = 74)	18 (24.3%)	5 (6.8%)	21 (28.4%)	44 (59.5%)
Mazandaran (*n* = 42)	7 (16.7%)	5 (11.9%)	16 (38.1%)	28 (66.7%)
Khouzestan (*n* = 69)	16 (23.2%)	5 (7.2%)	27(39.1%)	48 (69.6%)

*P < 0.5.

### Hemoplasmas in ectoparasites

In total, 91 dogs (24.6%; 95% CI 19.5–29.2) were infested with ectoparasites which were identified as *Ctenocephalides canis*, *Pulex irritans*, *Rhipicephalus sanguineus* sensu lato, *Heterodoxus spiniger*. Out of 210 PCR-positive dogs, 52 (24.8%) were infested with ectoparasites at the time of sampling (Table 1). Fleas collected from four dogs in Hamedan scored PCR positive using universal primers. Further testing with species-specific PCRs showed DNA of Mhc in *P. irritans*, CMhp in *P. irritans* and *C. canis*, and DNA of pathogens in one *C. canis* specimen. Except for the dog from which the latter flea was collected, corresponding dogs were PCR-positive. None of the examined ticks were found infected. In addition to fleas, ticks and lice, 11 *Hippobosca longipennis* keds were collected from 7 dogs (6 in Hamedan and 1 in Kermanshah) from which blood specimens could not be collected. However, all of the keds scored negative in PCR.

### Risk factors analysis for hemoplasma infection in dogs

Association between hemoplasma infection and potential risk factors are presented in Table 1. The presence of hemoplasma DNA was significantly higher in dogs older than 3-years-old. Furthermore, infection rate was higher in dogs with flea infestation and with poor body condition. There was no statistically significant association between positivity and gender or breed (Table 1).

### Sequence and phylogenetic analyses

**Figure 1:**
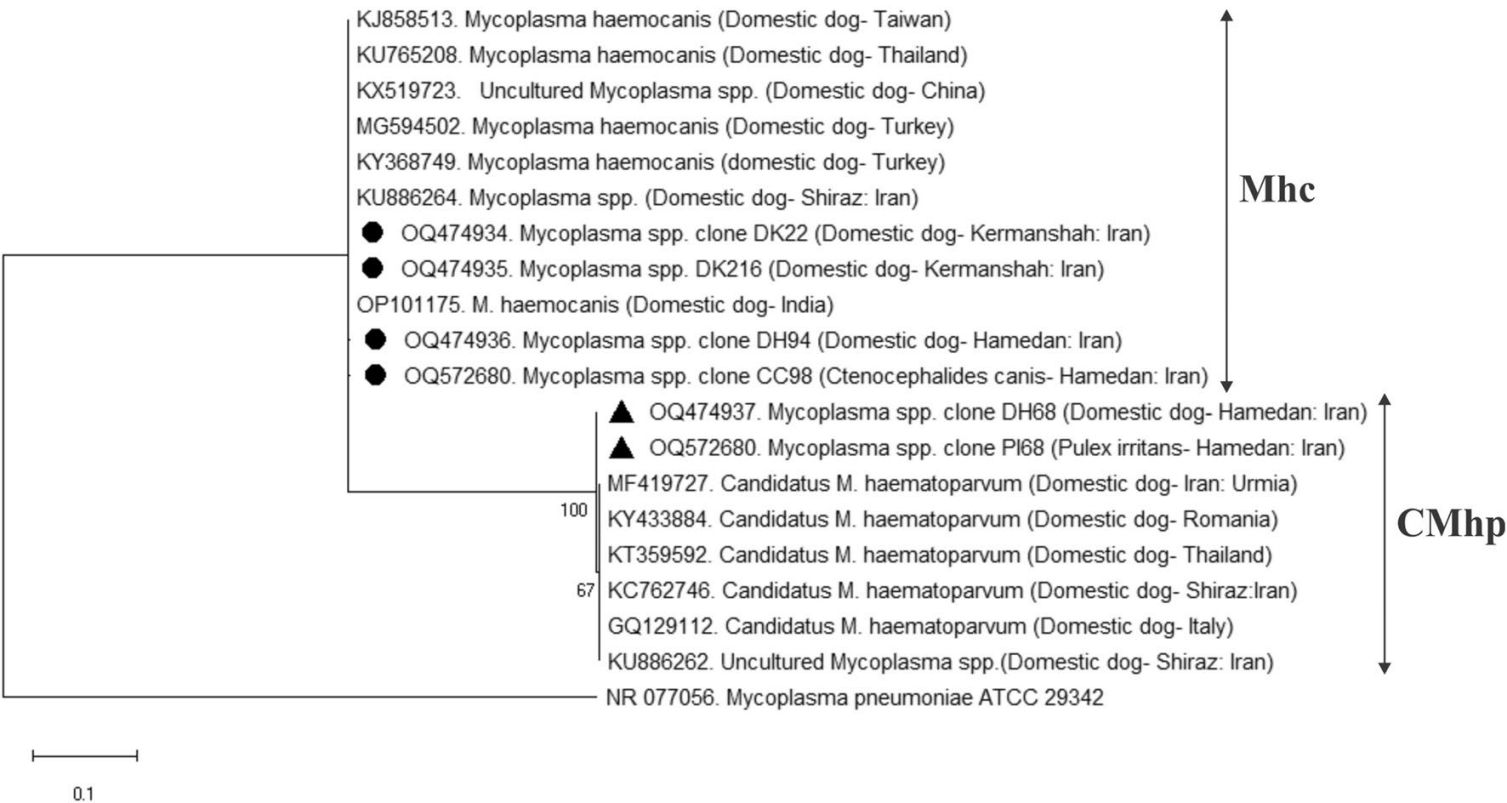
Phylogenetic relationship of *Mycoplasma haemocanis* (marked with circle) and *Candidatus* Mycoplasma haematoparvum (marked with triangle) sequences isolated in this study to other *Mycoplasma* spp. based on a partial sequence of the *16S rRNA* gene. The analyses were performed using maximum likelihood method. Homologous sequence from *Mycoplasma pneumonia* ATCC29342 (accession number: NR077056) was used as the outgroup. Mhc indicates the *Mycoplasma haemocanis* species and CMhp indicates *Candidatus* Mycoplasma haematoparvum species.

BLAST analysis of consensus sequences for detected pathogens displayed 99.8–100% nucleotide identity with Mhc and CMhp isolates available in GenBank® database. Representative sequences of pathogens detected in this study were deposited in the GenBank® database under the accession numbers OQ474934, OQ474935, OQ474936 for Mhc, OQ474937 for CMhp in dogs, OQ572680 for CMhp in *C. canis* and *P. irritans*, OQ572681 for Mhc in *P. irritans*. Molecular identification of nucleotide sequences for both organisms was supported by the distinct separation of species-specific clades inferred from the phylogenetic analyses (Fig. 1).

## Discussion

The high frequency of canine hemoplasmosis (56.8%) reported herein, indicates that apparently-healthy dogs from different regions in Iran are exposed to Mhc and CMhp, which poses health risks not only to canine but also people in contact with them. To our knowledge, the molecular prevalence found in this study is the highest infection rate reported in dogs to date although considerably high prevalences were previously reported in Australia (54%^24^), Brazil (44.7%^30^), Sudan (42.3%^31^) and, Portugal (40%^32^). Although both hemoplasma species were recorded in the country, previous studies reported much lower prevalence i.e. 10% in Shiraz^33^, and 26% in Esfahan^28^ which could potentially be due to environmental and climatic factors, veterinary care, and differences in the population structure (e.g. age) of the examined dogs. In particular, the infection was highest in dogs in Khuzestan (69.6%) and Mazandaran (66.7%) with hot-humid and Mediterranean climates, respectively, which favor the propagation of arthropod vectors. In neighboring Türkiye, 38% of sheltered dog^22^ and, in another study 15.3% of a mixed population stray, sheltered and domestic dogs^34^ were positive by PCR. In other studies in the region, 5.7% of stray dogs in Saudi Arabia were positive for Mhc^26^, 7.8% of client-owned dogs in Qatar^25^, and 17% of client-owned pet dogs in Egypt were positive for *Mycoplasma* spp.^27^. Present and previous reports suggest that apparently-healthy dog populations in West Asia and North Africa are chronically infected with hemoplasmas suggesting some level of enzootic stability against these pathogens. Since hemotropic *Mycoplasma* spp. including CMhp herein detected in 38.9% of dogs can infect humans^2^, raising the awareness of the public, persons in close relation with dogs (e.g. owners and veterinary personnel) and physicians about the risk of acquiring the infection from dogs is necessary. Moreover, since^30,35^ arthropods^36^ are still considered major players in the epidemiology of hemoplasmas, routine control of fleas and ticks is advocated. Finally, test-and-treatment of dogs even in the absence of relevant symptoms are strongly recommended.

In western Asia and north Africa, little information is available about the composition of hemotropic *Mycoplasma* species and genogroups infecting dogs. We found a higher prevalence of Mhc (17.83%) in comparison with CMhp (7.02%). Since the first record of CMhp in a dog in southern city of Shiraz^33^ few molecular-based studies have been performed. In Shiraz, 6 (6.0%) and 4 (4.0%) of 100 examined dogs were positive for Mhc and CMhp with no case of concurrent infection^29^. In another study in the same city, Mhc and CMhp were detected in 4 (7.5%) and 3 (5.7%) out of 53 dogs^37^. Similarly, examination of 100 dogs in central city of Esfahan showed higher prevalence of Mhc (13%) compared to CMhp (10%)^28^. In Türkiye also, infection of dogs with Mhc was higher than CMhp in two studies^22,34^. In contrast, CMhp was the dominant species infecting dogs in Egypt (15% vs 2% Mhc)^27^. However, it seems that co-infection with both species is common in dogs of the region i.e. 31.89% in this study, 3% in Esfahan Iran^28^, 5.3 and 6.4% in Türkiye^22,34^, and 2% in Egypt^27^ suggesting co-transmission of the pathogens.

Detection of Mhc in *P. irritans*, CMhp in *P. irritans* and *C. canis*, and simultaneous presence of pathogens in one *C. canis* indicates potential role of these fleas in the transmission of both pathogens in Iran although detection of pathogens in the body of fleas could be due to bloodmeal intake. However, statistically significant association between the presence of hemoplasma DNA in dogs’ blood and infestation with fleas, and simultaneous detection of hemoplasmas in three dogs and three fleas gives weight to the role of fleas in the transmission of hemoplasmas among dogs. The role of arthropod vectors in the epidemiology of hemoplasmosis is supported by the detection of canine and feline hemoplasma DNA in fleas collected from animals and/or the environment^1^. In particular, the cat flea, *C. felis*, has been implicated in feline hemoplasma transmission, but this has not been definitively proven in experimental studies^38^. Experimental studies on common flea species e.g. *C. canis*, *C. felis*, *C. orientis* and *P. irritans* could shed light on the competence of these fleas.

There are controversies regarding the role of ticks in the epidemiology of canine hemoplasmas. Only a single study has experimentally demonstrated transmission of Mhc by *R. sanguineus* s.l.^18^, and the role of this tick in the natural transmission of Mhc and other hemoplasmas is unknown. In a study in Switzerland, bacterial DNA was detected in *Ixodes* spp. and *Rhipicephalus* spp. ticks collected from animals, but not in unfed questing *Ixodes* spp. collected from vegetation^20,39^. Furthermore, higher prevalences of canine hemoplasmosis have been reported in countries and regions where *R. sanguineus* s.l. ticks are found more commonly^40^ hypothesizing that this tick may be a possible vector for the transmission of canine hemoplasma species. In contrast, none of 350 individual *R. sanguineus* ticks collected from dogs in a study in Türkiye where brown dog tick is prevalent^41^; and none of the tick specimens including 204 fully engorged nymphs removed from dogs, and 2100 nymphs and 85 adults collected from the grounds of the same shelter where both pathogens were present in examined dogs suggest that *R. sanguineus* s.l. may not be a suitable vector for canine hemoplasma species in the field conditions^22^. In the latter study, hemoplasma DNA was not detected in unfed adult ticks molted from engorged nymphs that had been collected from hemoplasma-infected dogs indicating that transstadial transmission did not occur in this tick species^22^. Thus, the importance of ixodid ticks including *R. sanguineus* s.l. in the transmission of canine hemotropic mycoplasmas remains unclear.

Relatively high prevalence of hemoplasmosis in dogs of this survey compared to previous studies might be associated with the examined population i.e. 98.1% were sheltered animals. In Iran, the animal shelters are mainly built by charity-based non-governmental organizations (NGOs) to feed stray dogs which are rescued from all over the province and protect them from culling program. Unfortunately, other than below-standards sanitation of these shelters, density of dogs is very high and thus posing dogs to stress and tension (AS personal observation) which could potentially lead to behavioral changes and aggressive interactions. Studies have successfully transmitted feline hemoplasma infection via subcutaneous inoculation of blood containing low numbers of organisms, suggesting possibility of pathogens transmission by fighting and biting in the field^42^. Interestingly, in a study in Cambodia transmission of haemotropic *Mycoplasma* was shown in the total absence of arthropod vectors in a closed population of dogs on ectoparasiticides^43^. In that study forty dogs were treated with two ectoparasiticide products and monitored for eight months. No new infections caused by *Babesia vogeli*, *Ehrlichia canis*, *Anaplasma platys*, and *Hepatozoon canis* which are proven to be vectorially-transmitted were detected; conversely, the number of haemoplasma infections in dogs rose significantly, providing strong evidence of non-vectorial transmission. Authors reported frequent dog aggression and fighting^43^ similar to the situation in Iran shelters. Other than possible transmission of hemoplasmas in fighting—when as little as 10 μl of cat blood infected with *Candidatus* Mycoplasma turicensis can produce an infection in a naïve cat^44^—DNA of *C. M. turicensis* was detected in the saliva and feces^20^, and DNA of *C*. M. haemominutum was detected in the salivary glands of infected cats^45^. Specific research on the transmission of canine haemoplasmas is crucial for establishment and implementation of methods to prevent new patients.

Association of infection rate and higher age of dogs is in agreement with the recent studies, which reported hemoplasmas to be more prevalent in older dogs^26,40,46^ and could be due to longer exposure time to pathogen. However, we found no significant association between hemoplasma infection rate and gender or breed, which is in accordance with previous reports in Cuba^47^, Nigeria^48^, Türkiye^34^ and Mediterranean countries^32^. Moreover, we found a significant association between hemoplasma positivity and body condition score of dogs i.e. 69.6% of dogs with poor body condition were PCR positive. This finding is somehow expectable since general health status of dogs is known to be linked with hemoplasmosis^34,49,50^.

There were some limitations in the present study such as limited number of pet dogs examined and number of Sanger sequenced samples because of resources. Moreover, hematological analyses could provide insightful information about the pathogenicity of strains since dogs in this study were asymptomatic. Additionally, future studies would benefit from typing the isolates using a panel of gene loci to shed light on possible differences in virulence and geographical distribution of strains. Finally, application of more sensitive and quantitative diagnostic methods e.g. real-time PCR will be beneficial not only in detecting more positive dogs but also to discover possible relationship between bacterial load and clinical manifestation.

## Conclusion

To our knowledge, this is the first large-scale molecular epidemiology study of canine hemoplasmosis in Iran. Considering the high prevalence of hemoplasmas in dogs all over the country including potentially zoonotic CMhp, routine ectoparasites’ control and test-and-treatment strategy especially prior to adopting sheltered dogs are recommended. Raising the awareness of the public, persons in close relation with dogs such as owners, shelters’ personnel and veterinary professionals, as well as physicians about the risk of acquiring the infection from dogs is also necessary. Last but not least, since recent findings suggest the transmission of canine haemoplasmas without involvement of arthropod vectors, establishment and implementation of novel methods to prevent their transmission is very much needed.

## Methods

### Ethical aspects

All applicable international, national, and institutional guidelines for the care and use of animals were followed. The blood and ectoparasites of dogs were collected with permission of the Ethical Committee of Hamadan University of Medical Sciences, Iran (code: IR.UMSHA.REC.1398.124) and the Ethical Committee of Bu-Ali Sina University, Iran (codes: IR.BASU.REC.1399.0014 and IR.BASU.REC.1399.0021). All methods reported in the present study are in accordance with ARRIVE guidelines (https://arriveguidelines.org).

### Study area and samples

**Figure 2:**
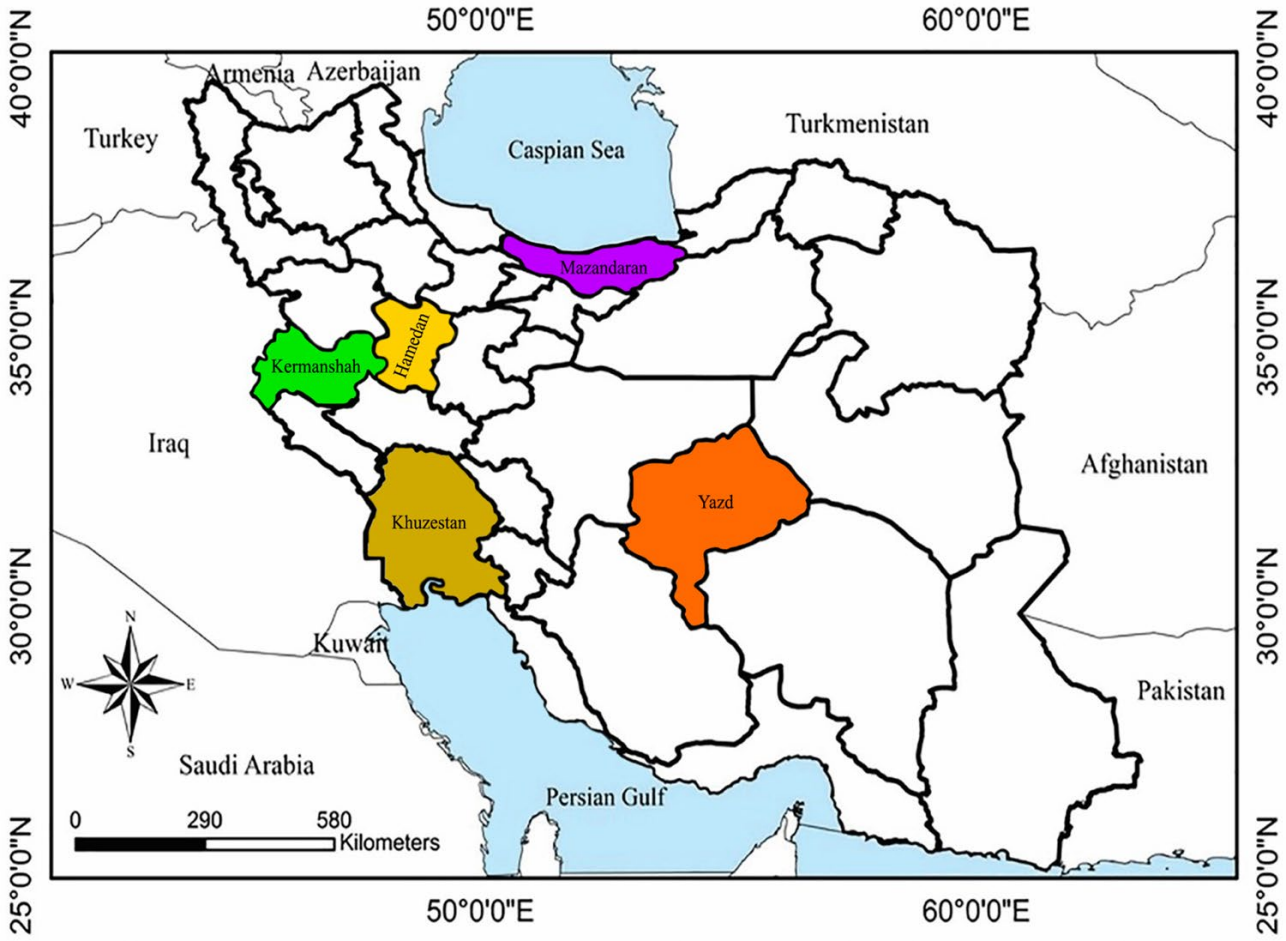
Samples were collected from five provinces in Iran. The map was drawn by using ArcGIS software version 10.3 (https://enterprise.arcgis.com/en/portal/).

From December 2018 to February 2021, cephalic or saphenous vein blood specimens were collected from 370 dogs in five provinces of Iran with different climates i.e. Hamedan (*n* = 135; cold semi‐arid) and Kermanshah (*n* = 50; warm and temperate) in the west, Yazd (*n* = 74; hot and arid) in the center, Khuzestan (*n* = 69; hot and humid) in the south-west and Mazandaran (*n* = 42; mild and humid) in the north (Fig. 2). Animal data i.e., gender, breed, age, keeping condition, body condition score and ectoparasites infestation were recorded. Blood and ectoparasite specimens were transferred to the Microbiology Laboratory of Faculty of Veterinary Medicine, Bu-Ali Sina University. Blood samples were kept in − 70 °C freezer until DNA extraction. Collected ectoparasites were counted and identified to species level using taxonomic keys^51,52^ at the earliest convenience then stored in separate microtubes in − 70 °C freezer until further molecular examination.

### DNA extraction

Genomic DNA was extracted from 200 µl of the blood samples using FavorPrep™ Genomic DNA Extraction Mini Kit (Favorgen, Pingtung, Taiwan) and from ectoparasites (ticks, fleas, keds) using Cell/Tissue WizPrep™ gDNA Mini Kit (Wizbiosolutions, Seongnam, South Korea). If more than one specimen belonging to one ectoparasite species was collected from a dog, specimens were pooled prior to DNA extraction. Quantity (260 nm absorbance) and quality (260/280 nm absorbance ratio) of extracted DNAs were measured by NanoDrop spectrophotometer (Thermo Scientific™ NanoDrop 2000, Waltham, MA, USA). DNAs were stored at − 20 °C until PCR screening.

### Molecular detections of the hemoplasma species

DNAs were initially screened by universal *Mycoplasma* spp. primers that amplifies a fragment of 16S rRNA gene^53^. Positive samples were further subjected to two species-specific PCR assays to determine the presence of CMhp and Mhc DNA using species-specific primers^54^. Primers sequences, expected amplicon sizes, and thermal cycling conditions are given in Table 2. PCRs were performed in a 25 µl volume reaction mixture consisting of 8 µl of distilled deionized water, 12.50 µl of Taq DNA Polymerase 2× Mastermix, 2.50 µl of the template DNA, and 1 µl of each forward and reverse primer. The concentration of primer was 10 pmol, except for CMhp-F and CMhp-R (30 pmol). PCR amplifications were performed in a SimpliAmp^TM^ thermal cycler (Thermo Fisher Scientific, Waltham, MA, USA). Positive DNA controls (CMhp & Mhc) that were kindly provided by Professor Dr. Roberta Iatta (University of Bari Aldo Moro, Italy), and distilled deionized water were used as positive and negative controls in each run.

**Table 2:** The oligonucleotide primers and PCR conditions used for molecular detection of the hemoplasma species.

Primer	Oligonucleotide sequence (5ʹ → 3ʹ)	Specificity	Target gene	Amplicon size (bp)	Cycling conditions	Ref.
HBT-F	ATACGGCCCATATTCCTACG	*Mycoplasma* spp.	16S rRNA	595	94 °C–10 min; (× 40) 95 °C–30 s, 62 °C–30 s, 72 °C–30 s; 72 °C–10 min	^44^
HBT-R	TGCTCCACCACTTGTTCA					
Mhc-F	GAAACTAAGGCCATAAATGACGC	*Mycoplasma haemocanis*	16S rRNA	309	94 °C–5 min; (× 32) 94 °C–1 min, 60 °C–1 min, 72 °C–1 min; 72 °C–5 min	^45^
Mhc-R	ACCTGTCACCTCGATAACCTCTAC					
CMhp-F	ACGAAAGTCTGATGGAGCAATAC	*Candidatus M. haematoparvum*	16S rRNA	328	94 °C–5 min; (× 37) 94 °C–1 min, 60 °C–1 min, 72 °C–1 min; 72 °C–5 min	^45^
CMhp-R	TATCTACGCATTCCACCGCTAC					

PCR amplified products were analyzed in 1% (w/v) agarose gel (SinaClon, Iran) stained with 0.5 µg/ml ethidium bromide (SinaClon, Iran) and electrophoresed at 110 V for 55 min. The gels were visualized and photographed using UV Camera and transilluminator (Vilber Lourmat, Collégien, France).

### Sequencing and phylogenetic analysis

Positive amplicons of *Mycoplasma* spp. from dog blood (*n* = 4) and fleas (*n* = 2) were Sanger sequenced in an Applied Biosystems 3500 Genetic Analyzer (Thermo Fisher Scientific, MA, USA) by Pishgam Biotech Company (Tehran, Iran). The sequencing results were first analyzed using Local Basic Alignment Tool (BLAST), edited by SnapGene^®^ software (GSL Biotech LLC, Chicago, USA), and then submitted to GenBank^®^ (NCBI) (www.ncbi.nlm.nih.gov). Alignments of obtained 16S rRNA sequence compared to the closely related sequences found in the GenBank^®^ database and phylogenetic tree construction were performed using Mega X (Molecular Evolutionary Genetics Analysis version 10)^55^, based on maximum likelihood method^56^. Evolutionary analyses were conducted on 1000 bootstrap replications.

### Statistical analyses

Association between the prevalence of canine hemoplasmas and potential risk factors i.e. age, breed, gender, sampling location, living condition, co-infections, and BCS of dogs were analyzed using a chi-square and Fisher’s exact test by SPSS version 26.0. (IBM Corp, Armonk, NY). *P-*values < 0.05 were considered as statistically significant differences.

## Data Availability

The datasets generated and analysed during the current study are available in the NCBI—GenBank—Nucleotide platform (https://www.ncbi.nlm.nih.gov/genbank/) and can be accessed through accession numbers: OQ474934, OQ474935, OQ474936 for *Mycoplasma haemocanis*, OQ474937 for *Candidatus* Mycoplasma haematoparvum in dogs, OQ572680 for *Candidatus* Mycoplasma haematoparvum in *Ctenocephalides canis* and *Pulex irritans*, OQ572681 for *Mycoplasma haemocanis* in *Pulex irritans*.
